# Thy-1^+^ Cancer-associated Fibroblasts Adversely Impact Lung Cancer Prognosis

**DOI:** 10.1038/s41598-017-06922-5

**Published:** 2017-07-25

**Authors:** Mark J. Schliekelman, Chad J. Creighton, Brandi N. Baird, Yulong Chen, Priyam Banerjee, Neus Bota-Rabassedas, Young-Ho Ahn, Jonathon D. Roybal, Fengju Chen, Yiqun Zhang, Dhruva K. Mishra, Min P. Kim, Xin Liu, Barbara Mino, Pamela Villalobos, Jaime Rodriguez-Canales, Carmen Behrens, Ignacio I. Wistuba, Samir M. Hanash, Jonathan M. Kurie

**Affiliations:** 1 Division of Public Health Sciences, Fred Hutchinson Cancer Research Center, Seattle Washington, United States of America; 2 Department of Medicine and Dan L. Duncan Cancer Center, Baylor College of Medicine, Houston, Texas United States of America; 3 Department of Bioinformatics and Computational Biology, University of Texas MD Anderson Cancer Center, Houston, Texas United States of America; 4 Department of Thoracic/Head and Neck Medical Oncology, University of Texas MD Anderson Cancer Center, Houston, Texas United States of America; 5 Department of Molecular Medicine and Tissue Injury Defense Research Center, Ewha Womans University School of Medicine, Seoul, Korea; 6 Department of Surgery, Houston Methodist Research Institute, Houston, Texas United States of America; 7 Department of Translational Molecular Pathology, University of Texas MD Anderson Cancer Center, Houston, Texas United States of America; 8 Department of Clinical Cancer Prevention, University of Texas MD Anderson Cancer Center, Houston, Texas United States of America

## Abstract

Cancer-associated fibroblasts (CAFs) regulate diverse intratumoral biological programs and can promote or inhibit tumorigenesis, but those CAF populations that negatively impact the clinical outcome of lung cancer patients have not been fully elucidated. Because Thy-1 (CD90) marks CAFs that promote tumor cell invasion in a murine model of Kras^G12D^–driven lung adenocarcinoma (Kras^LA1^), here we postulated that human lung adenocarcinomas containing Thy-1^+^ CAFs have a worse prognosis. We first examined the location of Thy-1^+^ CAFs within human lung adenocarcinomas. Cells that co-express Thy-1 and α-smooth muscle actin (αSMA), a CAF marker, were located on the tumor periphery surrounding collectively invading tumor cells and in perivascular regions. To interrogate a human lung cancer database for the presence of Thy-1^+^ CAFs, we isolated Thy-1^+^ CAFs and normal lung fibroblasts (LFs) from the lungs of Kras^LA1^ mice and wild-type littermates, respectively, and performed global proteomic analysis on the murine CAFs and LFs, which identified 425 proteins that were differentially expressed. Used as a probe to identify Thy-1^+^ CAF-enriched tumors in a compendium of 1,586 lung adenocarcinomas, the presence of the 425-gene signature predicted a significantly shorter survival. Thus, Thy-1 marks a CAF population that adversely impacts clinical outcome in human lung cancer.

## Introduction

In most tissues, fibroblasts are the primary stromal cells and are recognized by their spindle-shaped morphology and the absence of markers that define epithelial, immunologic, and other cell types. During wound healing and in pathological conditions such as fibrosis and cancer, fibroblasts acquire an activate phenotype with myofibroblastic features and, in cancer, are referred to as cancer-associated fibroblasts (CAFs)^1^. CAFs regulate the activities of immune cells, tumor cells, and endothelial cells, a multi-functionality that could result from the presence of multiple CAF lineages with distinct intratumoral functions. In support of this conclusion, lung cancer patient prognosis is improved by the presence of PDGFR-α/β^+^ CAFs and worsened by extracellular matrix (ECM) proteins commonly secreted by myofibroblastic CAFs^2–6^. Because factors secreted by CAFs can enhance tumor cell proliferation and invasion, promote angiogenesis, and suppress anti-tumor immunity, CAFs are an attractive target for cancer prevention and treatment^7^. Hence, studies are warranted to elucidate distinct CAF populations and their biological roles during tumorigenesis.

Thy-1 (CD90) is a heavily N-glycosylated cell surface protein that is expressed on a variety of tumor cells and normal cell types (e.g., fibroblasts, neurons, and endothelial cells) and has been shown to mediate cell-cell interactions by binding to integrins in a variety of tissue contexts^8, 9^. In the venous circulation, Thy-1 facilitates the attachment of tumor cells to endothelial cells and has been implicated in metastasis^10^. Thy-1 forms a tri-molecular complex with α5β1 integrin and syndecan-4 and supports β1 integrin- and syndecan-4-mediated mechanosignaling in melanoma cells^11^. In human lung cancers, a population of CAFs has been isolated that expresses Thy-1, α-smooth muscle actin (αSMA), and fibroblast activation protein (FAP), but fails to express CD45 and CD11b, a phenotype consistent with that of an activated myofibroblast, and elicits either a contact-dependent enhancement or suppression of tumor-associated T cell activation^12, 13^. Similarly, a Thy-1^+^ CD45^−^ Epcam^−^ population of CAFs has been isolated from Kras^LA1^ mice, which develop multifocal lung adenocarcinomas owing to somatic activation of a latent *Kras* ^*G12D*^ allele^14^; these CAFs exhibit biochemical and morphological properties of myofibroblasts, secrete diverse growth factors and pro-angiogenic and immunosuppressive cytokines^15^, and promote the invasive properties of lung adenocarcinoma cells derived from Kras^LA1^ mice in co-culture studies^15^. These features of CAFs are not due to somatic activation of the latent *Kras* ^*G12D*^ allele, which is in germline configuration in CAFs^15^. The lung parenchyma of wild-type mice and that of humans contain Thy-1^+^ lung fibroblasts (LFs)^16^. In contrast to CAFs, Thy-1^+^ LFs exhibit no myofibroblastic features or pro-invasive activity and secrete fewer cytokines in co-culture with tumor cells^15^. Thus, the biological properties of Thy-1^+^ CAFs and LFs are distinct, and additional studies are warranted to determine whether Thy-1^+^ CAFs promote lung tumorigenesis.

Here, we postulated that the presence of Thy-1^+^ CAFs confers a worse prognosis in human lung adenocarcinoma. To test this hypothesis, we initially examined the intratumoral location of Thy-1^+^ CAFs, which were found at the invasive front and in perivascular regions. Given that CAFs can generate gene expression signatures that have prognostic value in tumor transcriptomic studies^17^, we interrogated a human lung cancer database for the presence of Thy-1^+^ CAFs by performing global proteomic analysis on murine Thy-1^+^ CAFs and LFs, which identified 425 proteins that were differentially expressed. The 425-gene signature was used to probe a compendium of 1,586 lung adenocarcinomas, which showed that the presence of the 425-gene signature was correlated with a worse prognosis. These findings suggest that Thy-1 marks a CAF population that adversely impacts clinical outcome in human lung adenocarcinoma.

## Results

### Localization of Thy-1^+^ cells within tumor stroma

**Figure 1:**
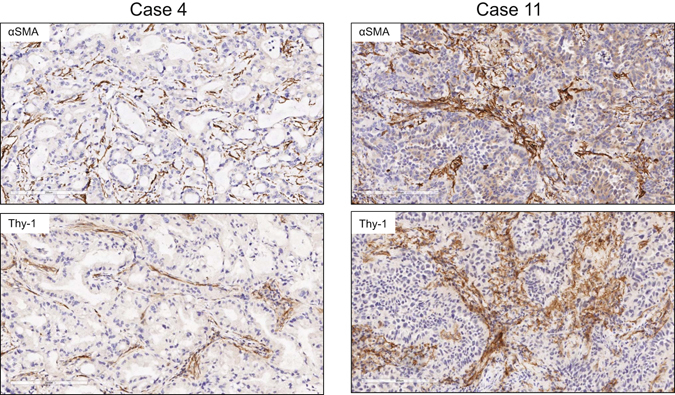
Detection of cells that express Thy-1 or αSMA in tumor stroma. Images of representative human lung adenocarcinomas with low levels (case 4) or high levels (case 11) of Thy-1 and αSMA. Tissues were subjected to immunohistochemical analysis and counterstained with hematoxylin. Cells that stain positively for Thy-1 or αSMA (brown) are located within stroma surrounding tumor cells (blue). Scale bar, 200 μm.

**Figure 2:**
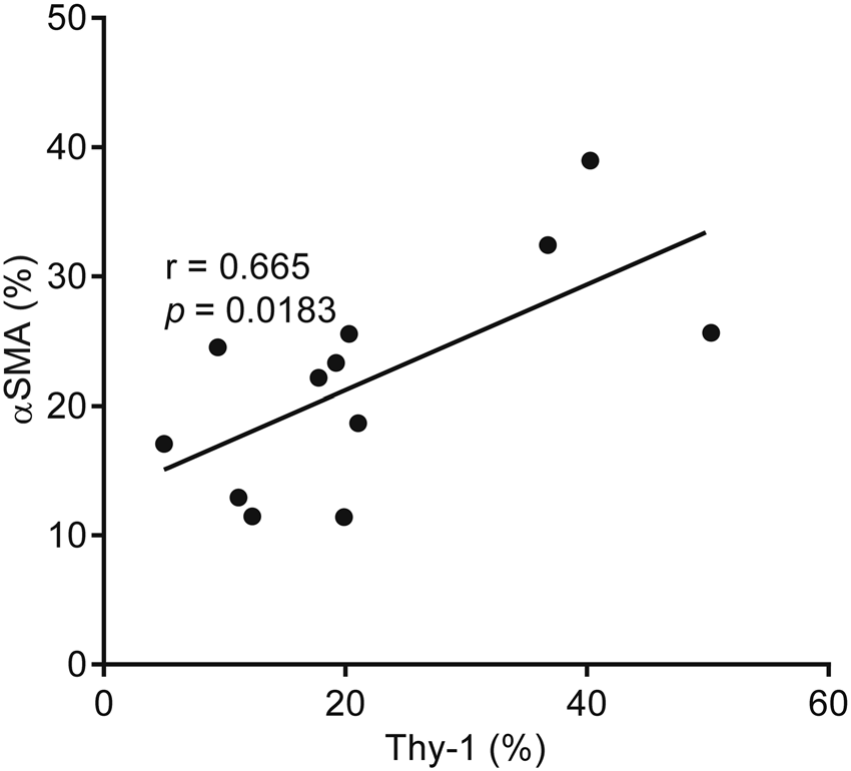
Positive correlation of Thy-1- and αSMA-expressing cells in tumor stroma. Scatter plot shows the percentages of cells per microscopic field per tumor that stain positively with anti-Thy-1 or anti-αSMA antibodies. Consecutive tissue sections were stained with anti-Thy-1 or anti-αSMA antibodies. Results represent mean values determined from multiple microscopic fields per tumor. Each dot represents a single tumor. P and r values, Pearson’s correlation analysis.

**Figure 3:**
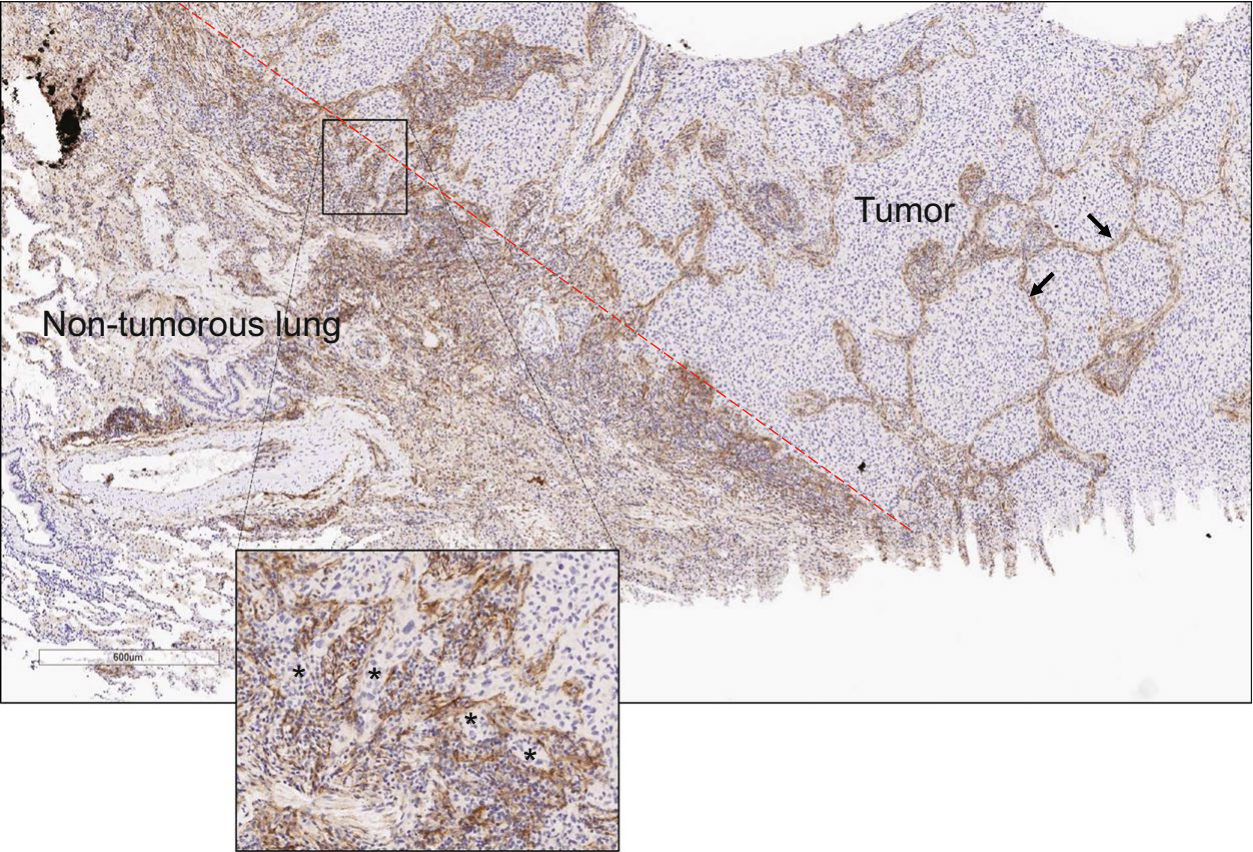
Thy-1-expressing cells localize within the tumor interstitium and on the tumor periphery. Image of human non-small cell lung cancer subjected to immunohistochemical analysis to detect Thy-1. Tissues counterstained with hematoxylin (blue). Cells that stain positively for Thy-1 (brown) are located within interstitial stromal bands (arrows) or at the boundary of tumor and non-tumorous lung tissues (dashed red line), a site of collectively invading tumor cells (asterisks, inset).

**Figure 4:**
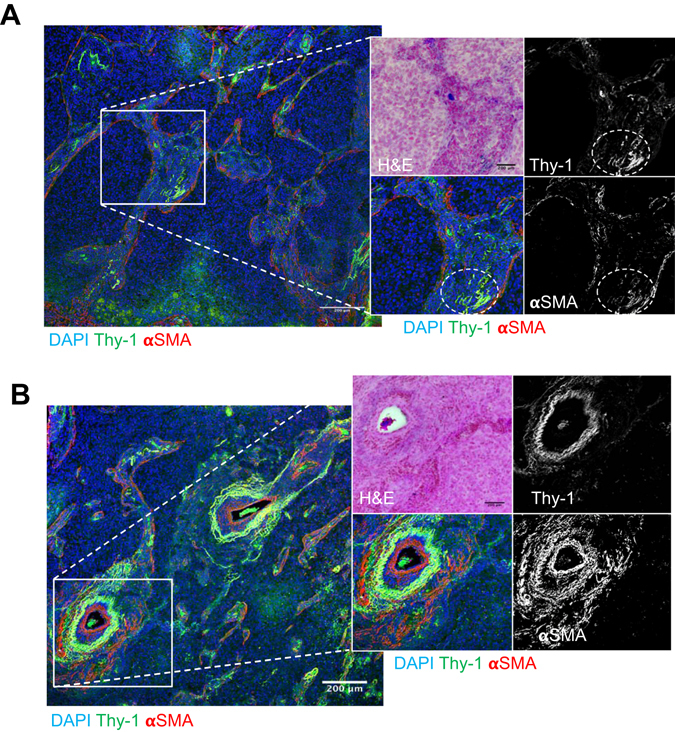
Stromal cells that co-express Thy-1 and αSMA. Merged fluorescence micrographs of human lung adenocarcinoma tissue sections co-stained with antibodies that detect Thy-1 or αSMA (pseudocolored green and red, respectively). Nuclei were counterstained with DAPI (pseudocolored blue). Illustrated at higher magnification (inset) are fluorescence and bright field micrographs of a stromal region (**A**) and a perivascular stromal region (**B**). The insets contain bright field image of tissues stained with hematoxylin and eosin (top left) and fluorescence micrographs of anti-Thy-1 and -αSMA shown as single channel images (grey scale, top and bottom right) and as merged images (pseudocolored, bottom left). Cells that co-express Thy-1 and αSMA are yellow. Regions of co-localization in Fig. 4A are indicated (ovals). Scale bar, 200 μm.

**Figure 5:**
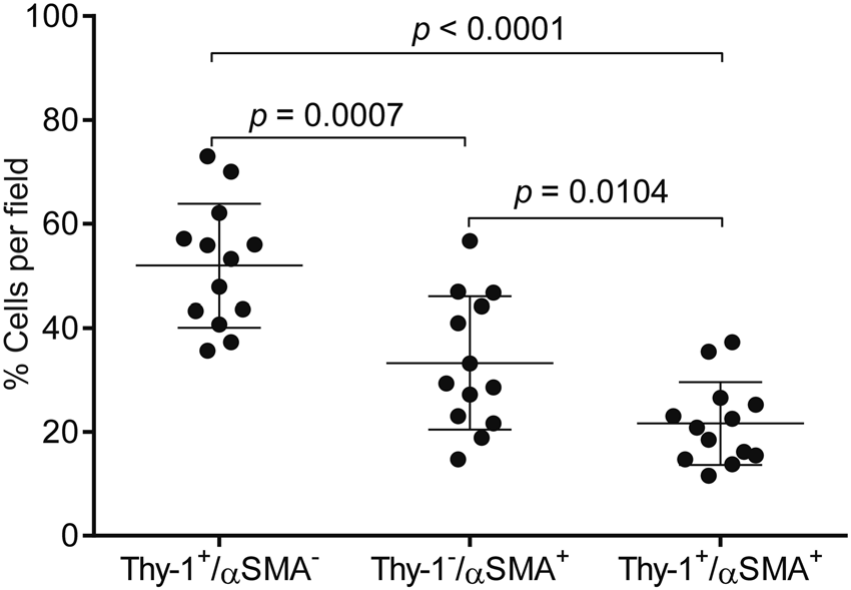
Quantification of cells that express Thy-1 or αSMA or both. Scatter plot shows the percentages of cells per microscopic field that are Thy-1^+^/αSMA^−^, Thy-1^−^/αSMA^+^, or Thy-1^+^/αSMA^+^. Each dot represents a single microscopic field from the same tumor specimen illustrated in Figs 4 and 5. A total of 13 randomly chosen microscopic fields were analyzed. Mean ± SD (bar and whiskers) from the 13 microscopic fields. P values, two tailed unpaired t test.

Flow cytometric studies have detected Thy-1-expressing (Thy-1^+^) CAFs in human lung cancers^13^, but their intratumoral location remains unclear. To address this question, we initially examined the intratumoral location of Thy-1^+^ cells and whether Thy-1^+^ cells co-express αSMA, a CAF marker. Thy-1 and αSMA were detected in tumor stroma by immunohistochemical analysis of human lung cancers (Fig. 1), and quantification of cells that express Thy-1 or αSMA within multiple microscopic fields showed that their levels were positively correlated (P = 0.018, r = 0.665) (Fig. 2). Thy-1^+^ cells were present in band-like stromal structures (arrows, Fig. 3) and at the invasive front on the tumor periphery (insets, Fig. 3). By confocal microscopic analysis, cells that expressed Thy-1 or αSMA or that co-expressed Thy-1 and αSMA were detected within stromal bands (Fig. 4A) and in perivascular regions consistent with a pericyte-like cell (Fig. 4B). By quantification, cells with a CAF phenotype (Thy-1^+^ αSMA^+^) accounted for approximately 20% of cells per microscopic field, whereas Thy-1^+^ αSMA^−^ cells (e.g., immunologic cells) and Thy-1^−^ αSMA^+^ cells (e.g., other fibroblast subsets) were significantly more numerous (Fig. 5). These findings support the presence of Thy-1^+^ CAFs within tumor stroma.

### An expression signature for the detection of Thy-1^+^ CAFs in tumors

To determine whether the presence of Thy-1^+^ CAFs impacts clinical outcome, we sought to probe publicly available lung cancer transcriptomic databases for evidence of Thy-1^+^ CAF-enrichment. Reasoning that a proteomic signature will exclude from the analysis those genes in the transcriptomic databases that are not differentially expressed at the protein level, we performed global proteomic analysis of matrix extracts prepared from Thy-1^+^ CAFs and LFs to identify those proteins that are up- or down-regulated in CAFs and then used that protein signature to probe transcriptomic databases.

**Figure 6:**
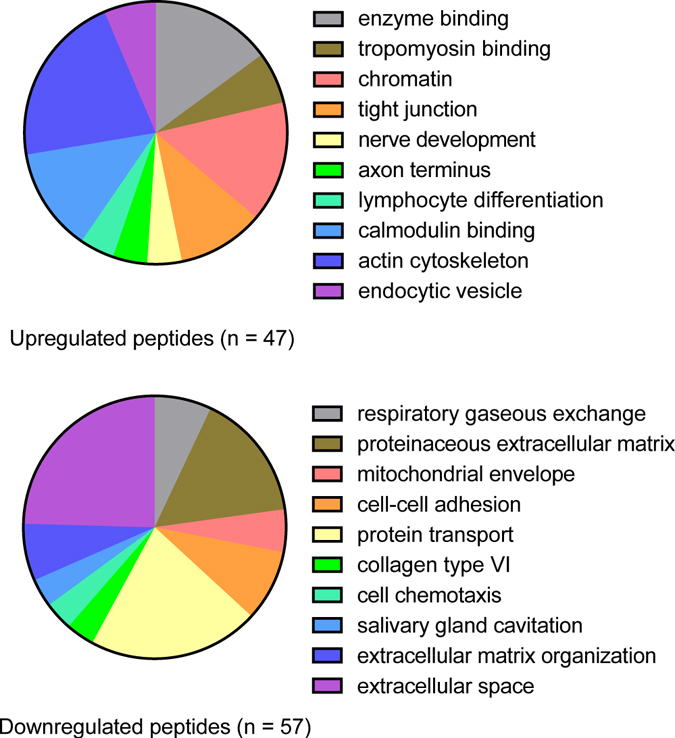
Gene Ontology term enrichment in the CAF proteomic signature. The 10 most highly enriched terms among peptides up-regulated in CAFs (top pie chart) or down-regulated in CAFs (bottom pie chart) relative to LFs. Pie segment size is proportional to the gene numbers represented in each term.

**Figure 7:**
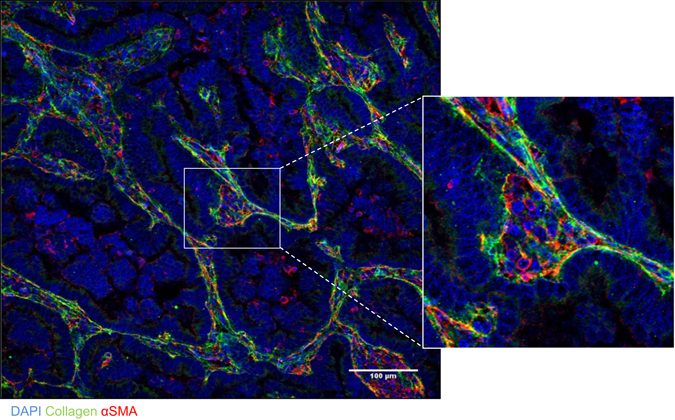
αSMA^+^ cells localize within collagenous stroma in a human lung adenocarcinoma. Merged fluorescence micrographs of a human lung adenocarcinoma tissue section co-stained with antibodies that detect type I collagen or αSMA (green and red, respectively). Nuclei were counterstained with DAPI (blue). Zoom (right) of boxed area shows a region of collagenous stroma that contains αSMA^+^ cells and surrounds papillary tumor structures.

Thy-1^+^ CAFs and LFs were isolated from the lungs of Kras^LA1^ mice and wild-type littermates, respectively, for the preparation of matrix extracts that were subjected to LC-MS/MS for comparison of proteomes by spectral counting. Of the 2,361 unique genes (2,441 IPI’s) identified (at least 2 peptides per gene), 1,197 genes (1,164 IPI’s) overlapped between CAFs and LFs, and 425 proteins were differentially expressed (G-Test > 3.9), including 223 upregulated and 202 downregulated proteins (Supplementary Tables 1 and 2). These included intracellular and secreted proteins, indicating that fibroblast matrix preparations were contaminated by the contents of lysed cells. The differentially expressed proteins were enriched in diverse Gene Ontology terms (Fig. 6). The up-regulated CAF peptides demonstrated enrichment in, among other terms, “enzyme binding” (e.g., IGF2R, BRCA1, PLAUR), “tropomyosin binding” (e.g., CNN3, TMOD3, CALD1), “chromatin” (e.g., HMGN1, HMGN2, PDS5A), and “tight junction” (e.g., MTDH, ARHGAP17, CXADR). To identify extracellular matrix molecules secreted by CAFs, we reanalyzed the proteomic profiling results using less strict criteria (G-test > 1.65) and identified multiple collagen isoforms (Col1a2, Col5a1, and Col5a3) that were secreted more abundantly by CAFs than LFs; this finding was validated by confocal microscopic analysis of a human lung adenocarcinoma, which showed papillary tumor structures surrounded by collagenous stroma containing αSMA^+^ cells (Fig. 7). The down-regulated peptides were enriched in, among other terms, “respiratory gaseous exchange” (e.g., NDFUA12, SFTPB, SFTPD), “proteinaceous extracellular matrix” (e.g., COL6A1, COL6A3, COL6A2, FBN1), “mitochondrial envelope” (e.g., TOMM20, HADHB, COX5B), “cell-cell adhesion” (e.g., COL6A2, VNN1, DSP, ITGA8, CDH1) and “protein transport” (e.g., ERC1, LRP1B, RRBP1, SANP23) (Supplementary Table 3). In addition, several basement membrane components were down-regulated, including basement membrane-associated proteoglycans nidogen-1 and laminin subunits LAMA5 and LAMC1. Thus, the CAF and LF proteomes contained widespread differences.

### A worse prognosis for lung adenocarcinoma patients with the Thy-1^+^ CAF expression signature

**Figure 8:**
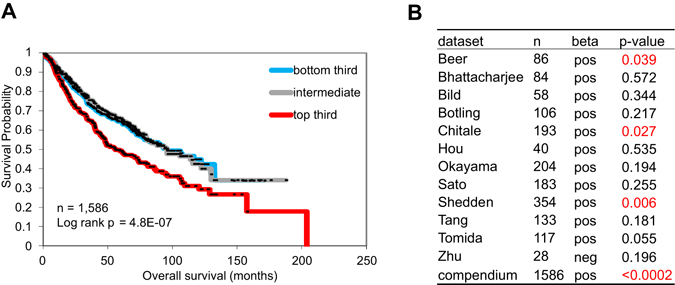
The presence of the CAF signature is correlated with a worse prognosis in human lung cancer. Survival analysis of lung adenocarcinoma patients, comparing the differences in risk between tumors, according to degree of manifestation of a CAF proteomic signature (in terms of corresponding mRNA patterns). Kaplan-Meier plot compares top third (“strong manifestation”), bottom third (“weak manifestation”), and middle third (“intermediate”) for a compendium of 12 independent lung adenocarcinoma cohorts. P-values by log-rank test. (**B**) Table shows correlations by univariate Cox, for each individual array dataset examined, as well as for a “compendium” of all available datasets (featured in Kaplan-Meier plot). Positive beta denotes correlation with worse patient prognosis.

Twelve independent cohorts of lung adenocarcinoma patients for which both gene expression and clinical outcome data were publicly available^18–29^ were scored based on the manifestation of the 425-gene signature in primary lung tumors, using a “t-score” metric as previously described^30^, where the overall t-score is high in a profile where the genes up in the CAF expression signature are relatively high while the genes down in the signature are relatively low. The association of the signature in lung tumors with patient survival was evaluated by both Kaplan-Meier plot (taking the top third, bottom third, and middle third of signature scores) and by univariate Cox (which evaluated the t-scores as a continuous measure, without the need for binning). We observed a correlation between the presence of the CAF signature and reduced overall survival duration (Fig. 8A), which reached statistical significance in three of the individual cohorts and as a “compendium” of all twelve cohorts (Fig. 8B); with the larger numbers providing greater power in the compendium dataset, clear survival trends may be evident that might have been missed in individual studies involving much fewer cases. This result indicates that the gene expression signature associated with CAFs is also associated with more aggressive lung adenocarcinoma in patients.

**Figure 9:**
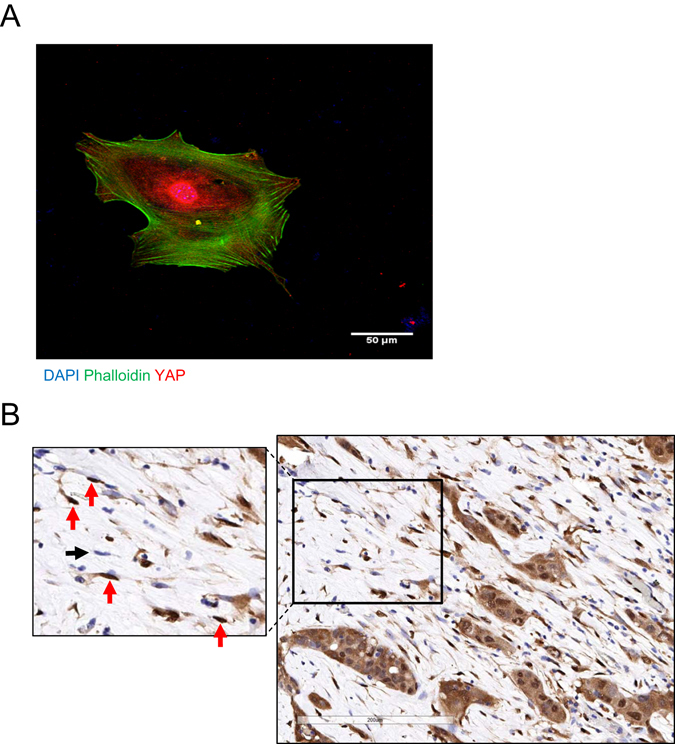
Nuclear YAP in stromal cells. (**A**) Merged fluorescence micrographs of a Thy-1+ CAF stained with anti-YAP antibodies (red), phalloidin (green), and DAPI (blue). (**B**) Anti-YAP immunohistochemical analysis of a stromal region within a human lung adenocarcinoma. Within the inset (left), arrows point to stromal cells with nuclei that stain positively (red) or negatively (black) for YAP.

By associating with integrins and syndecan-4 on adjacent cells, Thy-1 supports contractility-dependent mechanosignaling^31, 32^. External force activates the YAP and TAZ transcriptional regulators^33^, and YAP activation is required for CAFs to promote matrix stiffening, cancer cell invasion, and angiogenesis^34^. A hallmark of YAP activation, nuclear localization of YAP was detectable in 45 of 53 (84%) Thy-1^+^ CAFs grown in monolayer culture (Fig. 9A). Therefore, we postulated that CAFs have prognostic value because they generate mechanical signals that drive metastasis. To test this hypothesis, we performed immunohistochemical analysis of YAP expression on a cohort of early-stage lung adenocarcinoma patients (n = 248) that have been annotated on the basis of clinical outcome (Supplementary Table 4) and have a median survival duration of 67 months. We excluded tumor cells from the analysis and quantified YAP in regions of tumor stroma, where YAP was detectable in the cytoplasm and/or nuclei of a subset of stromal cells (Fig. 9B). A composite H-score was generated for cytoplasmic and nuclear YAP on the basis of staining intensity and extension, and the percentage of stromal cells deemed positive for nuclear YAP was quantified (Supplementary Table 4). By both metrics, nuclear YAP was not correlated with survival duration in this lung adenocarcinoma cohort.

## Discussion

Studies using experimental tumor models have established a strong link between CAF infiltration and the development of a tumor microenvironment that suppresses anti-tumor immunity, supports vascular growth, and promotes tumor cell invasion and metastasis^35^. Playing central roles in these processes are diffusible cytokines secreted by CAFs, such as vascular endothelial growth factor and transforming growth factor-β1^36^. However, CAFs are of heterogeneous origins, exhibit diverse biological properties, and can promote or inhibit tumorigenesis^37^. Tumor classification systems incorporating CAF expression signatures accurately predict patient outcome^2–5, 38–47^. Similar to the findings reported here, Navab *et al*. generated an expression signature from CAFs and LFs that had been isolated from human lung cancer and adjacent normal lung tissues, respectively, and found that the signature was correlated with a poor prognosis in human lung cancer cohorts^17^. However, the CAFs and LFs studied by Navab *et al*. were unselected and are probably more heterogeneous than the selected population (Thy-1^+^ CD31^−^ CD45^−^ Ep-CAM^−^) studied here. In fact, the signature reported here did not significantly overlap with that reported by Navab *et al*., suggesting that the cells are biologically distinct. Furthermore, the two studies undertook different expression profiling approaches (proteomic *versus* transcriptomic). The analysis undertaken here in which proteomic and transcriptomic databases were merged has inherent limitations if changes in the abundance of a transcript and the protein it encodes are not correlated. Regardless, proteomic signatures did reveal overlap with transcriptomic signatures in this study and in a separate report on colon cancer^48^.

The concept that resident fibroblasts can be converted to CAFs is supported by evidence that deregulated expression of specific microRNAs in resident ovarian fibroblasts supports CAF development in ovarian cancer^49^. Thy-1^+^ lung fibroblasts are considered progenitors of lung myofibroblasts in patients with idiopathic pulmonary fibrosis (IPF)^50^, but Thy-1 expression is silenced epigenetically in IPF-derived myofibroblasts^51^, suggesting that Thy-1 expression is not required for the maintenance of myofibroblast differentiation in IPF. Thy-1 is a cell surface glycoprotein that lacks a cytoplasmic domain but plays a critical role in contractility-dependent mechanosignaling^31, 32^. Given that mechanosignaling is required for the generation and maintenance of CAFs^34^, studies are warranted to examine whether Thy-1 drives CAF development in lung cancer.

Nuclear YAP expression in tumor cells is correlated with a worse prognosis in esophageal carcinoma, cervical carcinoma, hepatocellular carcinoma, gastric carcinoma, and lung cancer^52^. The findings reported here represent the first analysis, to our knowledge, of nuclear YAP specifically in tumor stroma. In contrast to the Thy-1^+^ gene expression signature, which was correlated with shorter survival in a subset of lung adenocarcinoma cohorts, nuclear YAP in tumor stroma was not correlated with a worse prognosis in a single patient cohort. Additional studies on independent cohorts are needed before definitive conclusions about the prognostic value of nuclear YAP in tumor stroma can be made, but the findings presented here raise the possibility that the prognostic value of CAFs is not related exclusively to mechanosignaling within tumor stroma.

## Methods

### Animal husbandry

Before their initiation, all mouse experiments were submitted to and approved by the Institutional Animal Care and Use Committee at the University of Texas MD Anderson Cancer Center, and all methods were performed in accordance with the relevant guidelines and regulations. Kras^LA1/+^ mice received standards of care and were euthanized according to the standards set forth by the IACUC. Kras^LA1^ mice have been maintained on their original 129sv background^14^.

### Immunohistochemistry and digital pathology analysis

Formalin fixed paraffin embedded tissue specimens from 12 patients with lung adenocarcinoma were selected for immunohistochemical staining to detect Thy-1 and αSMA. All cases were anonymized and approved by the Institutional Review Board. Five-μm-thick unstained sections from the cases were stained in a Leica Bond Max autostainer (Leica Biosystems, Nussloch GmbH). Slides were deparaffinized and rehydrated in the autostainer according to the manufacturer’s instructions. Antigen retrieval was performed with Bond Epitope Retrieval Solution #1 (Leica Biosystems), equivalent to citrate buffer pH 6.0 for detection of αSMA, and Bond Epitope Retrieval Solution #2 (Leica Biosystems), equivalent to EDTA pH 9.0, for detection of Thy-1. Primary antibodies employed were anti-αSMA (1:300, rabbit polyclonal, Abcam, catalog #ab5694), anti-Thy-1 (1:200, rabbit monoclonal clone D3V8A, Cell Signaling Technology, catalog #13801), anti-type I collagen (1:400, rabbit polyclonal, Abcam catalog #ab34710), and anti-YAP (1:100, rabbit monoclonal, clone D8H1X, Cell Signaling Technology, Danvers, MA, catalog number #14074). The antibodies were detected using Bond Polymer Refine detection kit (Leica Biosystems) and the labeling was visualized using 3′3′-diaminobenzidine and hematoxylin counterstaining. A human colon specimen was used as control for both Thy-1 and αSMA detection (cytoplasmic staining in the stromal cell compartment and smooth muscle cells, respectively). The staining quality was assessed by two pathologists. After staining, the slides were digitalized in an Aperio AT2 scanner (Leica Biosystems). The digital slides were then evaluated by a pathologist who selected five random areas of around 1 mm^2^. The analysis was performed on the selected tumor areas using a colocalization algorithm from the Aperio Image Toolbox analysis software (Leica Biosystems). The final data were expressed as the average of the percentage of tumor expressing Thy-1 or αSMA in the analyzed tumor regions.

For the quantification of YAP expression in a cohort of clinically annotated human lung adenocarcinomas, we used a tissue microarray containing 248 tumors (three core biopsies of one millimeter diameter per tumor). We employed 4-μm-thick sections of formalin-fixated, paraffin-embedded tumor blocks selected by a pathologist. Immunohistochemistry was performed using an automated staining system (Leica BOND-MAX; Leica Biosystems, Nussloch, GmbH) according to standard protocols. After incubation with the primary antibody, YAP expression was detected using a Novocastra Bond Polymer Refine Detection kit (Leica Bioosystems) with a diaminobenzidine reaction to detect antibody labeling and hematoxylin counterstaining. The immunostained sections were digitally scanned using the Aperio AT2 scanner (Leica Biosystems) under 20 × objective magnification. The images were visualized using ImageScope™ software (Aperio). The expression of YAP was evaluated in stromal cells using digital image analysis with Aperio Image Toolbox™ using a pathologist-trained nuclear and cytoplasmic-specific algorithm. YAP nuclear and cytoplasmic expression was evaluated according to the H-score system: The percent of staining intensity was scored as 0 (no staining), 1+ (weak staining), 2+ (moderate staining), and 3+ (strong staining), and the extension of the expression for each sample was reported as percentage of positive nuclei (0% to 100%). The final score (H-score) was then obtained by multiplying the intensity and reactivity extension values (range, 0–300) as we have reported^53^.

### Immunofluorescent staining

Immunofluorescent staining of human lung cancer tissue sections was performed as described elsewhere^54^. Bright field and fluorescence Images were acquired with a 20X (0.75 NA) objective on a Nikon A1+ confocal or with a 10X (0.45 NA) objective on a Nikon Eclipse Ti inverted widefield microscope (Nikon Corporation, Tokyo, Japan) equipped with DS-Qi2 monochrome camera (fluorescence) and DS-Ri2 color camera (color Brightfield) using NIS Elements software (Nikon). Raw images were deconvolved with Huygens Professional version 15.05 (Scientific Volume Imaging, The Netherlands, https://svi.nl/) and processed in ImageJ (NIH, Bethesda, https://imagej.nih.gov/ij). Briefly, deconvolved images were thresholded, smoothened and watershed segmented before creating a mask for DAPI channel to score for total number of cells per field. Other channels were processed identically and multiplied with the nuclear mask before scoring for number of single positive cells. Double positive cells were identified using RG2B colocalization plugin. Scores were expressed as percentage.

### Flow cytometric isolation of primary lung fibroblasts

CAFs and LFs were isolated from the lungs of Kras^LA1^ mice and wild-type littermates, respectively, by flow cytometric analysis to first remove hematopoietic cells (anti-CD45), endothelial cells (anti-CD31), and epithelial cells (anti-Epcam) and then positively select fibroblasts using an antibody against cell-surface glycoprotein Thy-1 (CD90). Briefly, murine lungs were digested into single cell suspension by immersion in 3 mg/mL of collagenase type I (Worthington, Lakewood, NJ) and 4 mg/mL dispase II (Roche, Indianapolis, IN) and mechanical mincing on a gentleMACS Dissociator (Miltenyi Biotec, Auburn, CA) followed by gentle rocking at 37 °C for 45 minutes. Dispersed cells were centrifuged, washed with PBS-2% fetal bovine serum, and subjected to red blood cell lysis (BioLegend, San Diego, CA). The remaining cells were centrifuged, washed, filtered (70 and 40 μm) and counted (Countess, Invitrogen, Grand Island, NY) for further processing. Fibroblasts were isolated by flow cytometry using directly conjugated monoclonal rat anti-mouse antibodies against CD31, CD45, Ep-CAM, and Thy-1 and relevant isotype controls tagged with fluorochromes including FITC, PE, PE-Cy7, or APC purchased from either BD Biosciences (Franklin Lakes, NJ), eBioscience (San Diego, CA), or Biolegend (San Diego, CA). 7-aminoactinomycin D (7-AAD; 2 μg/mL, Sigma Aldrich, St Louis, MO) was used as a viability dye for flow cytometric detection and exclusion of non-viable cells. Cells (5 × 10^7^ cells/mL) were resuspended in PBS and 2% FBS in an optimally pre-titered cocktail of antibodies and incubated for 45 minutes on ice. The labeled cells were washed and resuspended at 5–10 × 10^6^ cells/mL and held on ice for flow cytometric analysis and sorting using a FACSAria cell sorter (BD Biosciences). Fibroblasts were cultured in α-modified essential medium (MEM) (Corning, Manassas, VA) supplemented with 20% fetal bovine serum, penicillin/streptomycin, L-glutamine, and sodium pyruvate. Primary cells used in experiments had been passaged fewer than 6 times.

### Fibroblast matrix isolation

CAF and LF extracellular matrices were isolated based on a modification of previously published methods^55^. Briefly, fibroblast mono-cultures were grown to confluence in 150 mm plates in α-modified essential medium (Sigma Aldrich) supplemented with 20% fetal bovine serum, penicillin/streptomycin, 1X L-glutamine, and 1X sodium pyruvate. To enhance matrix secretion, L-ascorbic acid (50 µg/ml) was freshly prepared and added at each media change. For matrix isolation, cells were lysed in water for 10 minutes at which point cellular debris was removed. The remaining matrices (n = 3, each group) were scraped from plates and snap frozen for later analysis.

### Protein fractionation and liquid chromatography-mass spectrometry (LC-MS/MS)

Fibroblast matrices from LFs and CAFs (3 of each type) were prepared as described above. LC/MS-MS analysis was done as previously described^56^. In brief, samples were lysed first in a modified RIPA buffer (PBS, pH7.4; 0.1% SDS; 0.25% Na-deoxycholate) with phosphatase and protease inhibitors at 2 ml/g, followed by further extraction of the insoluble fraction with a urea lysis buffer (PBS, pH7.4; 5.0 M urea, 2.0 M thiourea; 0.1% SDS; 50 mM DTT). SDS and Na-deoxycholate was removed from the samples prior to fractionation with detergent removal spin columns (Pierce). Matrix proteins extracted as described above from fibroblasts were reduced overnight in DTT prior to fractionation. Individual fractions were trypsin digested and combined into 24 pools. Pools were then analyzed in a LTQ-ORBITRAP mass spectrometer (Thermo-Finnigan; San Jose, CA) with a nanoflow chromatrography system (Eksigent; Dublin, California). Acquired mass spectrometry data were automatically processed by the Computational Proteomics Analysis System (CPAS) pipeline using the X!Tandem search algorithm configured with the K-score module plug-in. The tandem mass spectra were searched against a composite database of IPI mouse (v3.64) and IPI bovine (v3.50). To estimate the significance of peptide and protein matches, we applied the tools of PeptideProphet and ProteinProphet. Peptides identified with PeptideProphet probability of a minimum 0.05 were selected and submitted to ProteinProphet; peptides with shared bovine sequence were not included. The derived protein identifications were filtered at a maximum 5% error rate. Peptide quantitation was computed by the Q3 quantitation tool using peptides achieving PeptideProphet probability of a minimum 0.5 and fractional delta mass not exceeding 20 ppm. Enrichment analysis for Gene Ontology terms was conducted on the differentially expressed proteins by Database for Annotation, Visualization, and Integrated Discovery (DAVID). GO categories with p-values > 0.01 and containing five or fewer genes from the list were discarded.

### Statistical analysis

Numerical values of cell culture and mouse cohort data were analyzed using student’s t-test for significance in GraphPad software. The difference was considered significant at P < 0.05 (two-tailed). For experimental data, all computations were carried out in SAS 9.2 and S-plus 8.0.

For analysis of mRNA expression and lung cancer patient survival, we examined a previously-assembled compendium dataset of 11 published expression profiling datasets for human lung adenocarcinomas (n = 1,492 tumors)^57–60^, with the addition of another dataset from Sato *et al*.^29^; patients represented in both Shedden and Chitale datasets (n = 88 patients) were first removed from the Shedden dataset, and one patient from Bild dataset thought to potentially represent squamous cell carcinoma was also removed (leaving n = 1,586 tumors in total). This compendium expression dataset was compiled using approaches previously described^60, 61^; briefly, genes within each array dataset were first normalized to standard deviations from the median; where multiple human array probe sets referenced the same gene, the probe set with the highest variation was used to represent the gene. In order to score each human lung tumor within the compendium dataset for similarity to our CAF proteomic signature, we derived a “t-score” metric for each human tumor in relation to the signature, using the same approach as that of previous studies^30, 58–60, 62–84^; briefly, the t-score was defined for each external profile as the two-sided t-statistic comparing, within the profile, the average of the genes high in CAF with the average of the genes low in CAF. From the 425 proteins in the CAF proteomic signature, 391 corresponding mRNAs (206 up in CAFs, 185 down in CAFs) were represented in the human lung tumor compendium.

## Electronic supplementary material

Supplementary Dataset 1

Supplementary Dataset 2

Supplementary Dataset 3

Supplementary Dataset 4
